# Electromagnetically Induced Transparency in Media with Rydberg Excitons 1: Slow Light

**DOI:** 10.3390/e22020177

**Published:** 2020-02-04

**Authors:** David Ziemkiewicz

**Affiliations:** Institute of Mathematics and Physics, UTP University of Science and Technology, Al. Prof. S. Kaliskiego 7, 85-789 Bydgoszcz, Poland; david.ziemkiewicz@utp.edu.pl

**Keywords:** electromagnetically induced transparency

## Abstract

In this paper, we show that Electromagnetically Induced Transparency (EIT) can be realized in mediums with Rydberg excitons. With realistic, reliable parameters which show good agreement with optical and electro-optical experiments, as well as the proper choice of Rydberg exciton states in the Cu_2_O crystal, we indicate how the EIT can be performed. The calculations show that, due to a large group index, one can expect the slowing down of a light pulse by a factor of about $10^{4}$ in this medium.

## 1. Introduction

In recent times, a lot of attention has been directed at the subject of excitons in bulk crystals due to the experimental observation of the so-called yellow exciton series in Cu_2_O, up to a large principal quantum number of n=25 [1,2,3,4]. In analogy to atomic physics, such excitons have been named Rydberg excitons (RE). By virtue of their specific properties, Rydberg excitons are of great interest in solid-state and optical physics. These objects, whose size scales as $n^{2}$ and where the energy spacing of neighbouring states decreases as $n^{-3}$, are well-suited for performing experiments in parameter ranges that are quite different from other quantum systems, such as atomic vapours. The small binding energy of RE makes them more sensitive to external magnetic and electric fields than other systems. The observation and detailed description of Rydberg excitons has opened a new field in condensed matter spectroscopy. Their specific properties have motivated both theoretical and experimental interest in this field, with studies ranging from their spectroscopy—that is, optical [2], electro-optical [3], and magneto-optical spectra [4,5] through non-atomic scaling laws [6] to quantum chaos [7].

In the last two years, particular interest has been directed to the study of the dynamical properties of systems with RE. Due to a large orbital radius, their dipole moments are exceptionally large for higher values of *n*, so that the interaction between higher Rydberg excitons is exceptionally strong and leads to the so-called Rydberg blockade. The appearance of a Rydberg blockade separates linear and nonlinear regimes of dynamical phenomena with RE. In the nonlinear regime, the possibility of observing giant optical nonlinearities of RE in microcavities has been considered [8], while the paper [9] deals with strong interaction phenomena at very low densities of RE, which enables one to determine the contribution of the nonlinear optical response of the medium. In the case of a smaller exciton density, it is possible to remain in the linear range, for which a single-photon source has been proposed [10]. RE mediums could be used as a gain system for solid-state, highly tunable masers, allowing to achieve output powers of order ranging from $10^{-6}$ to $10^{-2}$ W in continuous and pulse mode, respectively [11]. The realization of light slowing or storing and retrieving experiments in crystal with RE could unlock a plethora of dynamical effects, which might be observed in such media. These phenomena are based on the electromagnetically induced transparency (EIT), which has various potential applications in both classical and quantum optics. Słowik et al. [12] theoretically proposed to implement single-qubit gates and a two-qubit controlled not (CNOT) gate operating on polarized photons based on light storage. Recently, a highly efficient quantum memory protocol based on EIT has been experimentally demonstrated for cold rubidium [13] and cesium atoms [14]. Experimental evidence of spinor slow light as a quantum memory for two-color qubits in cold rubidium has also been presented [15].

EIT [16] is one of the most important effects utilized in quantum optics as it allows for the coherent control of materials’ optical properties. The generic EIT utilizes extraordinary dispersive properties of an atomic medium with three active states in the Λ configuration. This phenomenon leads to a significant reduction of absorption of a weak, resonant laser field (probe field) by irradiating the medium with a strong control field, and thus making an otherwise opaque medium transparent. This leads to dramatic changes of dispersion properties of the system. Absorption forms a dip called the transparency window and approaches zero, while the dispersion in the vicinity of this region becomes normal with a steep slope, which increases for a decreasing control field—the resonant probe beam is transmitted almost without losses. It should be stressed that there is a significant distinction between Electromagnetically Induced Transparency and Autler–Townes Splitting (ATS) [17]. The Autler–Townes effect results in a “split” transition within a coupled three-level system due to the AC Stark effect and emerges in the strong coupling limit of the more recently discovered electromagnetically induced transparency [17]. The EIT effect is described by the formation of a dark state due to a destructive quantum interference between the transition pathways. Both ATS and EIT result in a transparency feature, which is qualitatively identified as a wide spectral region between the split-absorption peaks for ATS, but a narrow transmission window within a single-absorption peak for EIT. This common feature has been at the centre of long-standing confusion as to whether an observed transparency is due to EIT or ATS, and as such, the distinction between the two is an active topic of research in the quantum optics community. It has recently been shown theoretically [18], as well as experimentally [19] that it is possible to objectively distinguish between the regime where EIT dominates (EIT regime) and the one where ATS dominates (ATS regime). Usually, EIT is explained by the destructive quantum interference between different excitation pathways of the excited state, or alternatively, in terms of a dark superposition of states. For at least 20 years, there has been a considerable level of activity devoted to the EIT [20], which was motivated by the recognition of a number of its potential applications, such as slowing and storing the light (see e.g., [21,22,23] and the References therein). Some of the most exciting developments on EIT refer to their manifestations in artificial atoms [24,25,26,27]. A remarkable quenching of absorption due to EIT in an undoped bulk crystal of Cu_2_O in a Λ configuration involving lower levels was examined in [28], and evidence of quantum coherences has been pointed out to exist in Cu_2_O [29].

In Rydberg systems, a ladder configuration enables one to couple long-living metastable, initially empty upper levels with the lower levels coupled by the probe field. Demonstration of EIT in Rydberg atoms involving the ladder levels scheme by Mohapatra et al. [30] has taken advantage of their unique properties and entailed investigations concerning transient and steady-state EIT spectra in Rydberg systems [31]. The Rydberg excitons offer an unprecedented potential to study the above-mentioned phenomena in solids—excitons in Cu_2_O offer a great variety of accessible states for creating the ladder configuration, which enables such a choice of coupling that could be realized by accessible lasers, or which could eventually to be suitable for desirable coherent interaction implementations. The first step toward the study of the photon blockade, many-body physics with Rydberg excitons and quantum non-linear optics is the realization of Rydberg EIT. The optical nonlinearity can be significantly enhanced in the presence of quantum interference in EIT systems, so examination of this phenomena in RE may be the first step to proposing an eventual application of this medium in quantum information processing.

Here, we focus our interest on the dynamical aspects and properties of Rydberg excitons in Cu_2_O, and we show that the Rydberg excitonic states can be used to realize the EIT. We indicate excitonic states which guarantee the most efficient realization of the experiment, taking into account our previous results concerning excitonic resonances [2,3,4,5], as well as damping parameters and the matrix elements of the interband dipole operator. The influence of nonlinear interactions will also be taken into account. Moreover, a study of Rydberg EIT spectra will be performed in the presence of dipolar interactions, and their dependence on excitonic density, principal quantum numbers of Rydberg states, and control beam intensities will be discussed.

Our paper is organized as follows. In Section 2 we present the assumptions of the considered model and solve the time evolution equations, obtaining an analytical expression for the susceptibility and the group index. We use the obtained expression to compute the real and imaginary part of the susceptibility and the group index (Section 3) for a Cu_2_O crystal slab. We examine in detail the influence of the control field on the dispersion, absorption, and the group index of the medium. In Section 4, we draw conclusions based on the model studied in this paper and indicate the optimal choice of Rydberg states to realize the EIT and light slowing in the linear regime.

## 2. Theory

The phenomenon of EIT, described qualitatively above, has been studied theoretically in various configurations of the transitions, probe, and control beams. Below, we propose a theoretical description for the case when the atomic transitions are replaced by intra-excitonic transitions in Rydberg excitons. The condensed matter exhibits quite a variety of three-level systems where an induced transparency could be achieved in much the same way as done in atomic mediums. Yet, dephasings, which can easily break the coherence of the population trapping state, are typically much faster in solids than in atomic vapours; it has caused great difficulty in observing a large, electromagnetically induced transparency effect in solids. We believe that this difficulty could be overcome by using the Rydberg exciton states. In particular, the higher states have exceptionally long lifetimes (which are proportional to $n^{2}$ [1]) and the dephasing can reach values which enables observation of the EIT effect.

**Figure 1:**
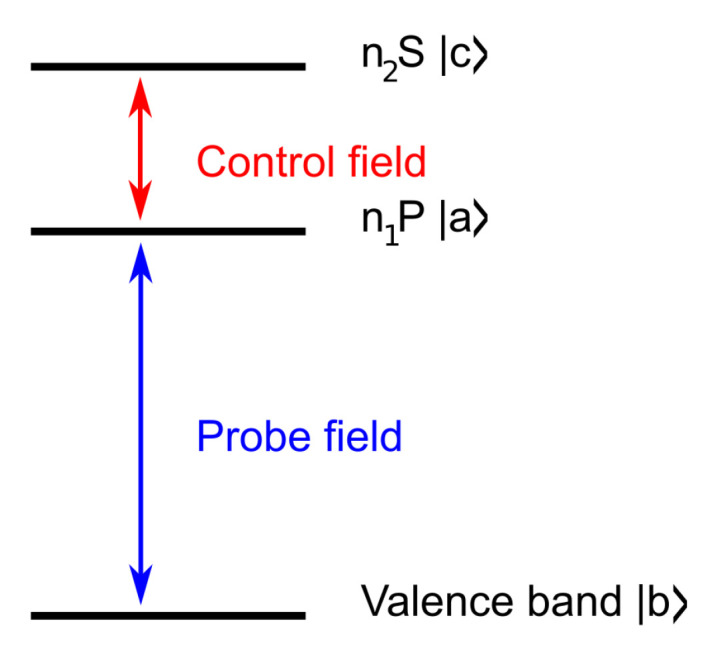
Schematic of the considered ladder EIT system.

Below, we consider a Cu_2_O crystal as a medium where the EIT phenomenon can be realized. We use a ladder configuration (Figure 1) consisting of three levels, a,b, and *c*. As in previous works on Rydberg excitons, we focus our attention on the so-called yellow series associated with the lowest inter-band transition between the $\Gamma_{7}^{+}$ valence band and the $\Gamma_{6}^{+}$ conduction band. Because both band-edge states are of even parity, the lowest 1S exciton state is dipole-forbidden, whereas all the *P* states are dipole-allowed; the 1S to nP transition is also allowed. We have chosen the valence band as the *b* state. As a practical example, the $n_{1}P$ and $n_{2}S$ excitonic states are chosen. The proposed ladder system contains only dipole-allowed S→P transitions.

Let the probe/signal field of frequency $\omega_{1}$ and amplitude $\varepsilon_{1}$ couple the ground state *b* of energy $E_{b}$ with an excited state *a* of energy $E_{a}$. The control field of frequency $\omega_{2}$ and amplitude $\varepsilon_{2}$ couples the state *c* of energy $E_{c}$ with the state *a*, as it is illustrated in Figure 1. The Hamiltonian of such a three-level system interacting with an electromagnetic wave in the rotating wave approximation leads to

(1) $$\begin{array}{ccc} H & = & E_{a}\left|a\rangle \langle a\right|+E_{b}\left|b\rangle \langle b\right|+E_{c}\left|c\rangle \langle c\right|\\ & & +\left\{-\hbar \Omega_{1}\left(z,t\right)\exp\left[-i\left(\omega_{1}t-k_{1}z\right)\right]\left|a\rangle \langle b\right|\right.\\ & & \left.-\hbar \Omega_{2}\left(z,t\right)\exp\left[-i\left(\omega_{2}t-k_{2}z\right)\right]\left|a\rangle \langle c\right|+h.c.\right\}+V_{nl},, \end{array}$$

where h.c. stands for hermitian conjugate. The $k_{1}$, $k_{2}$, $\Omega_{1}\left(z,t\right)=\left(1/\hbar \right)d_{ab}\varepsilon_{1}\left(z,t\right)$, $\Omega_{2}\left(z,t\right)=\left(1/\hbar \right)d_{ac}\varepsilon_{2}\left(z,t\right)$ are the wave vectors and real Rabi frequencies corresponding to the particular couplings, respectively. The $d_{ij}$ are the dipole transition moments related to the specific transitions, and $V_{nl}$ describes the nonlinear interactions between Rydberg excitons. One of the characteristic features of Rydberg media is the existence of the Rydberg blockade. Strong dipolar interaction between Rydberg excitons, which strongly depends on their distance, shifts the Rydberg levels, preventing the optical excitation of nearby excitons by shifting their corresponding levels out of the resonance with the exciting electromagnetic field. The term Rydberg blockade refers to the case where the interaction-induced shift is much larger than the EIT linewidth. In this case, the resulting nonlinearity can be interpreted as a switch from three-level EIT susceptibility to two-level susceptibility [32]. Resonant absorption and exciton creation are no longer possible inside the blockade volume $V_{bloc}$, in which dipole interaction energy is larger than the EIT linewidth. This effect influences the generic, linear EIT, so that the nonlinear modification should be taken into account [32]. In the presented manuscript, we consider the situation when one remains in the linear regime, that is, for exciton density small enough to avoid the Rydberg blockade. The presented calculations allow one to make such a choice of excitonic states where linear approximation still holds, such as the blockade-induced shift $\delta_{Rydb}/\Gamma_{ab}<<1$; this is ensured by the exciton density which is two to three orders of magnitude below the limit where the excitons are packed closely together and $\delta_{Rydb}∼\Gamma_{ab}$.

The time evolution of the system is governed by the von Neumann equation with a phenomenological relaxation contribution

(2) $$i\hbar \frac{d\sigma }{dt}=\left[H,\sigma \right]+R\sigma ,$$

where σ(z,t) denotes the density matrix for an exciton at position *z* and time *t*, and *R* is the relaxation operator accounting for all relaxation processes in the medium.

By neglecting propagation effects for the control field and denoting the probe and control beam detunings by $\delta_{1}=\left(E_{a}-E_{b}\right)/\hbar -\omega_{1}$ and $\delta_{2}=\left(E_{c}-E_{a}\right)/\hbar -\omega_{2}$, respectively, the evolution of the system can be described by the following equations:

(3) $$\begin{array}{ccc} i{\dot{\sigma }}_{aa} & = & -\Omega_{1}\left(\sigma_{ba}-\sigma_{ab}\right)-\Omega_{2}\left(\sigma_{ca}-\sigma_{ac}\right)-i\Gamma_{ab}\sigma_{aa}+i\Gamma_{ca}\sigma_{cc},\\ i{\dot{\sigma }}_{bb} & = & \Omega_{1}\left(\sigma_{ba}-\sigma_{ab}\right)+i\Gamma_{ab}\sigma_{aa}+i\Gamma_{cb}\sigma_{cc},\\ i{\dot{\sigma }}_{cc} & = & -\Omega_{2}\left(\sigma_{ac}-\sigma_{ca}\right)-i\Gamma_{ca}\sigma_{cc}-i\Gamma_{cb}\sigma_{cc},\\ i{\dot{\sigma }}_{ab} & = & \left(\delta_{1}-i\gamma_{ab}\right)\sigma_{ab}-\Omega_{1}\left(\sigma_{bb}-\sigma_{aa}\right)-\Omega_{2}\sigma_{cb},\\ i{\dot{\sigma }}_{bc} & = & \left(\delta_{2}-\delta_{1}-i\gamma_{cb}\right)\sigma_{bc}+\Omega_{2}\sigma_{ba}-\Omega_{1}\sigma_{ac}+\sum_{j\neq i}V_{ij}\sigma_{cc}^{j}\sigma_{bc}^{i},\\ i{\dot{\sigma }}_{ca} & = & \left(\delta_{2}-i\gamma_{ca}\right)\sigma_{ca}-\Omega_{2}\left(\sigma_{cc}-\sigma_{aa}\right)-\Omega_{1}\sigma_{cb}+\sum_{j\neq i}V_{ij}\sigma_{cc}^{j}\sigma_{ca}^{i}.. \end{array}$$

At large separations between RE, the dipole–dipole interaction is dominant, and the potential $V_{ij}$ strongly depends on the separation distance between Rydberg excitons [1]. The parameters $\Gamma_{ij}$, i≠j describe the damping of exciton states and are determined by temperature-dependent homogeneous broadening due to phonons, and broadening due to structural imperfections and eventual impurities. The relaxation damping rates for the coherence are denoted by $\gamma_{ij}\approx \Gamma_{ij}/2$, i≠j [28]. It should be noted that in the above equations, only the relaxations inside the three level system are considered, so that the total probability for the populations of the three levels is conserved: $\sigma_{aa}+\sigma_{bb}+\sigma_{cc}=1$.

Due to the fact that at t=0, $\sigma_{bb}\left(t=0\right)=1$ for a weak probe field (i.e., $|\Omega_{1}{|}^{2}<<|\Omega_{2}{|}^{2})$, in the first-order perturbation with respect to the probe field, the evolution of our system, given by Bloch equations, reduces to a set of the following equations for the density matrix:

(4) $$\begin{array}{ccc} i{\dot{\sigma }}_{ab} & = & \left(\delta_{1}-i\gamma_{ab}\right)\sigma_{ab}-\Omega_{1}-\Omega_{2}\sigma_{cb}, \end{array}$$

(5) $$\begin{array}{ccc} i{\dot{\sigma }}_{bc} & = & \left(\delta_{2}-\delta_{1}-i\gamma_{cb}\right)\sigma_{bc}+\Omega_{2}\sigma_{ba}+\sum_{j\neq i}V_{ij}\sigma_{cc}^{j}\sigma_{bc}^{i}. \end{array}$$

Taking into account the fact that for a weak probe pulse, for a given frequency, the polarization of the medium $P_{1}$ is proportional to the signal field ε and susceptibility χ, it takes the form

(6) $$P_{1}=\epsilon_{0}\chi \left(\omega_{1}\right)\varepsilon_{1}\left(\omega_{1}\right)=Nd_{ba}\sigma_{ab},$$

where *N* is the density of excitons, $d_{ba}$ is the transition dipole matrix element [11], and $\epsilon_{0}$ is the vacuum dielectric permittivity. In the limit of the low probe intensity, the susceptibility, which follows from the steady-state solutions of Equations (4) to (5), can be expanded into the Taylor series

(7) $$\begin{array}{c} \chi \left(\omega_{1}\right)=\chi^{\left(1\right)}+\chi^{\left(3\right)}\Omega_{1}^{2}=-\frac{N|d_{ab}{|}^{2}}{\hbar \epsilon_{0}}\frac{1}{\omega_{1}-\delta_{1}+i\gamma_{ab}-\frac{|\Omega_{2}{|}^{2}}{\omega_{1}-\delta_{1}+\delta_{2}+i\gamma_{cb}}}+\chi^{\left(3\right)}\Omega_{1}^{2}, \end{array}$$

where $\chi^{\left(1\right)}$ is the linear, and $\chi^{\left(3\right)}$ is the nonlinear part of electric susceptibility. The formula for the linear part $\chi^{\left(1\right)}$ follows from the steady-state solution of Equations (4) to (5) and $\chi^{\left(3\right)}$ is treated as a correction, which depends on interaction potential $V_{ij}$ and causes the nonlinear modification of EIT due to the strong interactions between Rydberg excitons. For a large exciton separation *R*, this potential has the form $\;C_{6}/R^{6}$, with $C_{6}$ being a coefficient proportional to $n^{11}$, which drastically increases for higher Rydberg excitons. On the other hand, for closer separation of excitons, when the dipole–dipole interaction becomes comparable or even exceeds the energy spacing between excitonic levels, this interaction has the Foerster type and is proportional to $n^{4}/R^{3}$ [1]. In our system, we remain in the low-density regime where the $\;C_{6}/R^{6}$ interaction is dominant.

The susceptibility is a complex, rapidly varying function of $\omega_{1}$, with its real part responsible for the dispersion and the imaginary part describing the absorption. If the excitons are driven on a single-photon resonance $\delta_{1}\approx 0$, the main nonlinear effect will be the nonlinear absorption. Neglecting the transverse probe beam dynamics for lower excitonc states, that is, when van der Waals is dominant, it is possible to analytically calculate this nonlinear part of susceptibility [33],

(8) $$\chi_{Re}^{\left(3\right)}=\frac{4\sqrt{2}\pi^{3}\gamma_{ab}\Omega_{2}^{4}C_{6}{\left|C_{6}\right|}^{-1/2}}{k^{3}\sqrt{\gamma_{ab}}{\left(\gamma_{ca}\gamma_{ab}+\Omega_{2}^{2}\right)}^{7/2}}N^{2};\;\chi_{Im}^{\left(3\right)}=\left|\chi_{Re}^{\left(3\right)}\right|.$$

It should be stressed that the interaction between Rydberg excitons modifies the system in a nonlinear way by shifting the Rydberg levels, effectively changing the control field detuning and influencing the absorption and the width of the transparency window. Note that the equality of real and imaginary parts of $\chi^{\left(3\right)}$ holds in a limited frequency range around the a→b resonance [33].

Due to the dependence of the refractive index n(ω1)=ϵb+Reχ(ω1) on the medium dispersion, we define the group index

(9) ng(ω1)=c/vg=1+12Reχ(0)+ω12∂∂ω1Reχ(ω1),

which accounts for the time delay of a pulse propagating in a medium with the group velocity

(10) vg=c1+12Reχ(0)+ω12∂Reχ(0)∂ω1.

The slope of dispersion relation inside the transparency window determines the velocity of the pulse propagating inside the medium. Note that the derivative of the real part of the susceptibility may be positive (normal dispersion) or negative (anomalous dispersion); in the latter case, the group velocity may even become negative.

## 3. Numerical Results

We considered a Cu_2_O crystal slab with a thickness of 30 μm as a medium where the EIT phenomenon can be realized. We used the ladder configuration (Figure 1), consisting of three levels. Let the probe/signal field of frequency $\omega_{1}$ couple the ground state (valence band) with the excited state $n_{1}P$. The control field of a much lower frequency $\omega_{2}$ couples states $n_{1}P$ and $n_{2}S$. Using the Formulas (7) and (9), we calculate the real and imaginary part of the susceptibility and the group index. For the state energies and damping parameters, we use the values obtained from fitting to experimental data, as described in [3]. The transition dipole moments were calculated from hydrogen-like functions, as described in detail in [11]. In particular, we used Hydrogen P-orbital with the radius proportional to $n^{2}$ [1], which results in dipole moments in the range of 1.5 ea${}_{b}$ (1S state) up to over 150 ea${}_{b}$ (10P state) [11]. Note that instead of $d_{ba}$, we used the function $M_{01}\left(r\right)$, which is an analogue of the dipole moment element, but takes into account the smeared-out transition dipole density characteristic for coupling between the valence band and the 2*P* excitonic state [4]. Application of this smeared-out dipole density is justified by a good agreement with an experiment of our previous results for electro-optical properties of Rydberg excitons [3]. We chose the exciton density $N=2.5\cdot 10^{14}\;{\mathrm{cm}}^{-3}$, which is two orders of magnitude below the maximum value of $N∼\frac{3\cdot 10^{18}}{n_{1}^{7}}\approx 2.6\cdot 10^{16}$ cm^−3^ for $n_{1}=2$ [1].

The system parameters are summarized in Table 1. We assumed that $\gamma_{ij}=\Gamma_{ij}/2$ [28]; the damping rates are calculated for T=10 K, where the contribution of exciton scattering with acoustic phonons is relatively small, and the main factor is spontaneous, with an emission rate proportional to $n^{3}$ [11].

**Figure 2:**
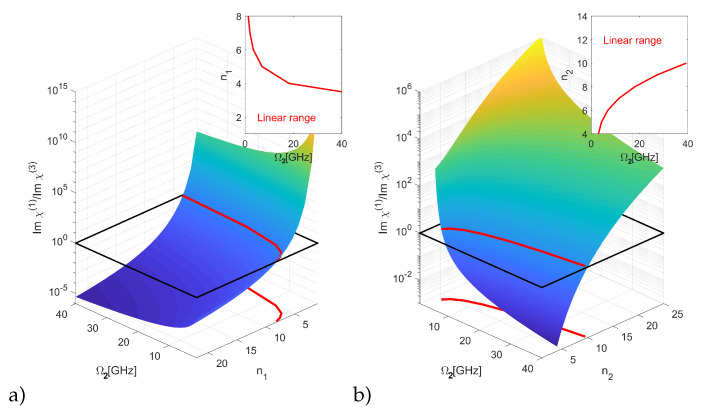
The ratio of the linear to nonlinear susceptibility as a function of control field strength and state number (**a**) $n_{1}$ and (**b**) $n_{2}$; colors added for clarity. Inset: the range of parameters where the linear susceptibility is larger.

The choice of states $n_{1}$ and $n_{2}$ has a fundamental effect on the properties of the system. The interaction coefficient $C_{6}∼n_{1}^{11}$ shows the strongest dependence on the state number and puts an upper limit on the lower state $n_{1}$. Moreover, the maximum exciton density is inversely proportional to the Rydberg blockade volume $V_{bloc}∼n_{1}^{7}$ (note that the upper state $n_{2}$ is empty, so that the blockade does not apply to it). The state $n_{2}$ affects the susceptibility by relaxation rates. All these factors indicate that a successful light slowdown can be achieved only for some parameters and the state configurations, where the linear part of susceptibility is dominating, and thus the group velocity is well-defined. Therefore, as a first step, we have investigated the range of states and control field intensities where the linear part of susceptibility is dominating, as shown in the Figure 2. As pointed out above, the nonlinearity is very sensitive to the state number and varies by many orders of magnitude. One can also see that, in general, linear response is obtained for weaker control fields, which is advantageous for a large slowdown factor, but results in a relatively narrow transparency window. In the range where $\chi^{\left(3\right)}$ is negligible, there is very little absorption inside the transparency window, and the group velocity $V_{g}$ is a well-defined quantity that can be calculated in a linear regime from Equation (11).

The linear region is outlined on the insets of the Figure 2. One can see that, practically, only the lower states $n_{1}$ = 1, … 4 are usable, except for extremely small values of control field intensity which are impractical due to the fact that in EIT, the control field needs to be much stronger than the probe field, that is, $\Omega_{2}>>\Omega_{1}$. On the other hand, the $n_{2}$ should be large; due to the fact that this level remains empty, it is not constrained by the Rydberg blockade.

**Figure 3:**
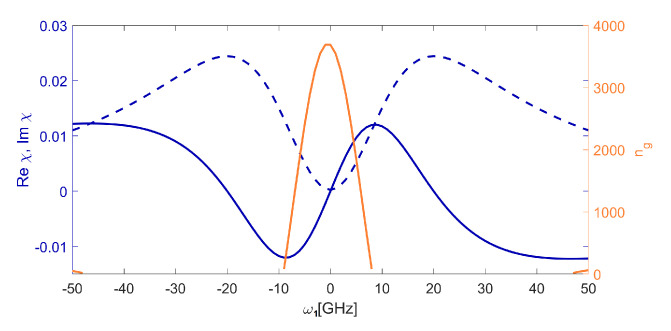
The real and imaginary parts of linear susceptibility and group index as a function of detuning, for $n_{1}=2$, $n_{2}=10$, $\Omega_{2}=10$ GHz, $\Omega_{1}=1$ GHz.

**Figure 4:**
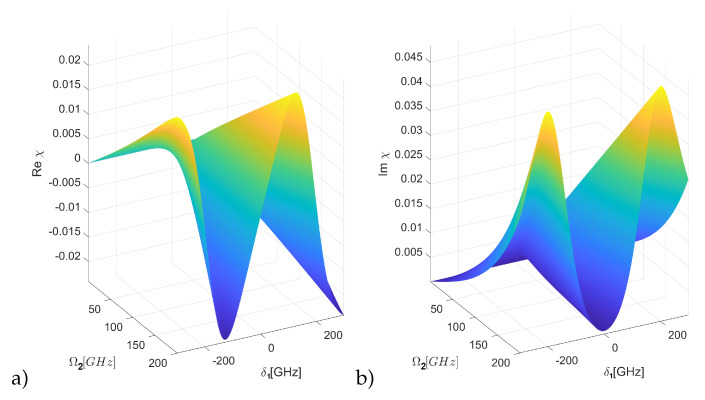
The real (**a**) and imaginary (**b**) part of the linear susceptibility as a function of control field strength and frequency for $n_{1}=2$, $n_{2}=10$, $\Omega_{1}$ = 0.1$\Omega_{2}$. Colors added for clarity.

The Figure 3 shows the medium susceptibility and the group index $n_{g}=c/V_{g}$. For a non-zero Rabi frequency $\Omega_{2}$ of the control field, the imaginary part of the system’s susceptibility reveals a dip in the Lorentzian absorption profile, called a transparency window. This means that the resonant probe beam which otherwise would be strongly absorbed is now transmitted almost without losses. The width of the transparency window is proportional to the square of the control field amplitude, and therefore, by increasing the control field strength, it is possible to open it out. This is shown in the Figure 4, where one can observe a dip in absorption (Im χ) around the zero-detuning part of the spectrum, which is accompanied by a very steep slope of the Re χ. That strong, normal dispersion inside the window is responsible for the reduction of the group velocity. The absorption at the resonance does not reach zero due to the finite value of the relaxation rate $\gamma_{cb}$, but it is indeed very small (absorption coefficient α∼ 3 cm^−1^). This is easily visible in the Figure 3, where the control field is set to $\Omega_{2}=10$ GHz. This means that the medium has become almost transparent for a probe pulse which now travels with a reduced velocity. Indeed, the group index shows that the impulse slowdown factor of about 3700 is expected. It should be stressed that the value of $n_{g}$ is directly proportional to the magnitude of susceptibility changes, and thus, to the exciton density. Clearly, high values are favourable; for the lowest state, the densities on the order of $10^{19}$ cm^−3^ are possible [34,35]. In our calculations, we have chosen $N=2.5\times 10^{14}$ cm^−3^, which corresponds to susceptibility values on the order of 0.03 (Figure 3), comparable with values presented in [28] for the low excitonic states and well below the limit imposed by the Rydberg blockade for the chosen state. It should be stressed that while these densities are orders of magnitude greater than in atomic EIT systems, the possible impulse slowdown is limited by much larger dissipation rates, particularly $\gamma_{ab}$.

**Figure 5:**
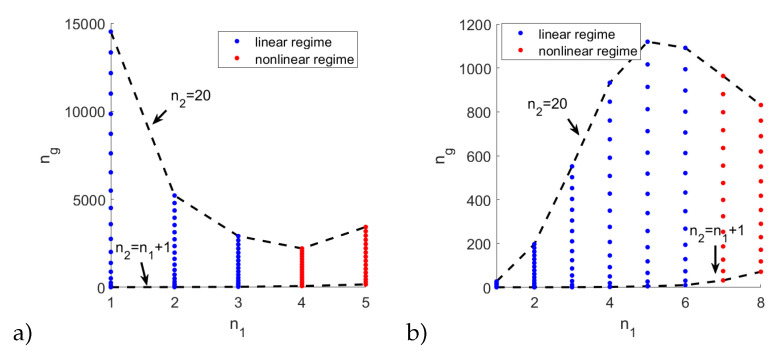
Maximum group velocity slowdown as a function of $n_{1}$ and $n_{2}$ for (**a**) $\Omega_{2}=30$ GHz (**b**) $\Omega_{2}=3$ GHz.

As mentioned previously, there are many Rydberg state combinations that provide a working EIT system. The maximum possible slowdown in all sets of states $n_{1}=1,\ldots 10$, $n_{2}=2,\ldots 20$, a strong control field $\Omega_{2}=30$ GHz, and probe field $\Omega_{1}=3$ GHz is shown in the Figure 5a. The blue dots mark the systems where the linear part of susceptibility is much greater than the nonlinear, so that the group velocity is a well-defined quantity. The red dots mark the range where Reχ<Imχ, so that the calculated slowdown is only a rough estimation. Remarkably, a very large slowdown can be achieved for $n_{1}=1$ due to the small size of excitons and, correspondingly, large exciton density. In the strong control field regime, the linear systems can be based only on $n_{1}=1,2,3$; for higher states, the effect of a Rydberg blockade becomes significant. Moreover, it is beneficial to use the high upper state $n_{2}$ so that the probe and control field frequencies are similar, for example, $\omega_{1}\approx \omega_{2}$. It should be pointed out that a high value of $n_{2}$ is only possible at highly cryogenic temperatures. Additionally, the probe beam may excite a group of closely-lying upper states, further degrading the performance [11].

By reducing the probe and control field strengths to $\Omega_{1}=0.3$ GHz and $\Omega_{2}=3$ GHz, correspondingly, one can extend the range of linear systems to $n_{1}=6$, as shown in the Figure 5b. In such a case, the transparency window is much narrower. Due to this, the slowdown in the systems with $n_{1}<5$ is limited by the large dissipation constant $\gamma_{ab}$, which prevents the transparency window from fully opening. On the other hand, the weaker probe and control fields result in a smaller density, so that the Rydberg blockade effect is delayed to $n_{1}>6$.

## 4. Conclusions

The Rydberg excitons are now hopefully emerging as a tool for quantum technology due to their unique properties, allowing for easy state selection and strong interaction with applied electromagnetic fields. Their unusual features can be useful for controlling matter–electromagnetic field interactions, which offers a new approach for studying semiconductor systems and also provides entirely new long-term perspectives for developing novel devices, which are more robust and compact than atomic systems. We have indicated the optimal states and well-justified parameters to attempt the observation of EIT in Rydberg excitons’ Cu_2_O media, which allows one to obtain a considerable value of the group index. Due to the coherence properties of Rydberg excitons, the manipulations of the medium transparency is possible; the width of the window and slowing down the group velocity of the pulse travelling inside the sample might be changed in a controlled way by the strength of the control field. The ability to control the group index on-demand enables one to store and retrieve light pulses, which is a first step toward quantum memory implementation. The method of precise dynamical control of the optical properties of the medium by optical means reveals new aspects of excitonic quantum optics and is supposed to lead to constructing an efficient tools for photonics, quantum switches, or multiplexers. Since the first observation of Rydberg excitons in 2014, their spectroscopic properties have mostly been investigated. Only a few observations have dealt with quantum optical aspects; the quantum coherence effects have been analyzed in [29], and a Rydberg exciton single-photon source has been proposed [10]. These studies have just opened up the possibility for a controlled light-matter coupling in a GHz regime with RE media. So far, the experimental demonstration of EIT in Rydberg excitons media has not been accomplished yet, but one may expect such experiments in the future. Performing the EIT in excitonic Rydberg media will be a step towards realization of controlled interaction of Rydberg excitons in integrated and scalable solid-state devices and potential implementation of this medium for quantum information processing.

## Figures and Tables

**Table 1:** Parameters used in calculations.

Parameter	
$\omega_{1}$	3291 THz
$\omega_{2}$	31.71 THz
$\gamma_{ab}$	4.558 THz
$\gamma_{bc}$	36.46 GHz
$\Gamma_{ab}$	9.116 THz
$\Gamma_{bc}$	72.92 GHz
$\Omega_{2}$	20 GHz
*N*	$2.5\cdot 10^{14}\;{\mathrm{cm}}^{-3}$
$M_{01}$	$2.1\cdot 10^{-30}$ C·m
